# Letter from the Editor in Chief

**DOI:** 10.19102/icrm.2023.14086

**Published:** 2023-08-15

**Authors:** Moussa Mansour


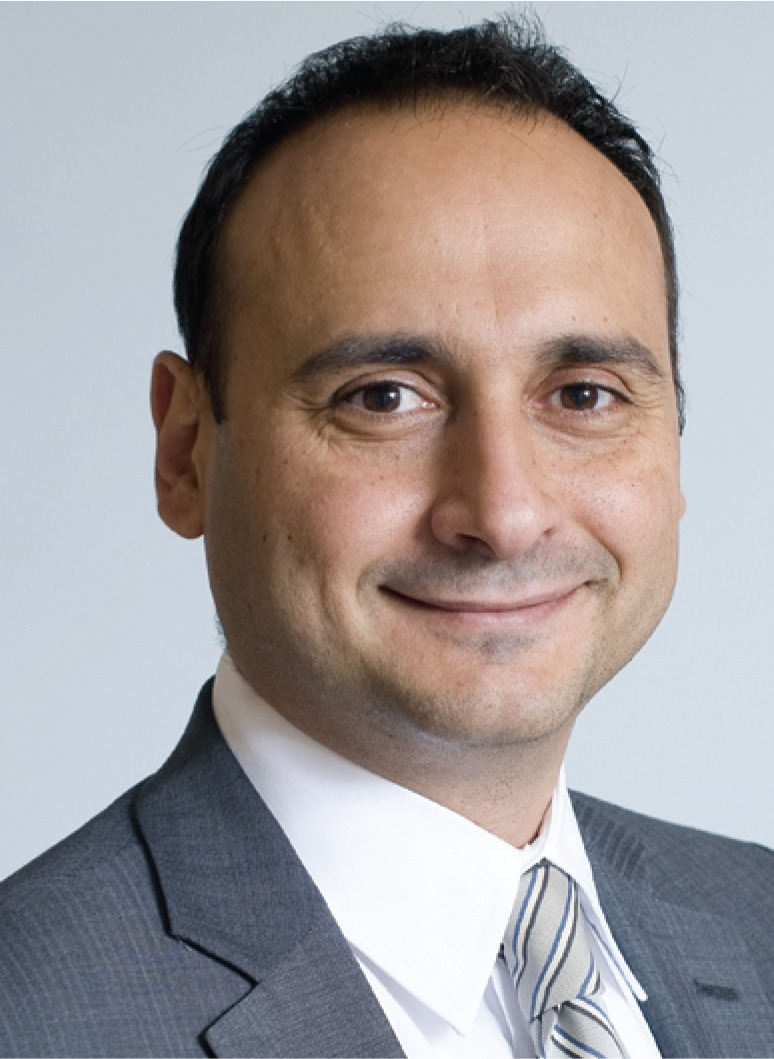


Dear readers,

The impact of obesity on the prevalence of atrial fibrillation (AF) has been extensively investigated. Many studies have also examined the relationship between obesity and outcomes of AF ablation and demonstrated that obesity is associated with lower success and higher complication rates during AF ablation. As a result of these data, many AF centers developed multidisciplinary programs combining weight loss with AF treatment to maximize the chance of restoring sinus rhythm.

This issue of *The Journal of Innovations in Cardiac Rhythm Management* contains an interesting article tackling the other end of the spectrum of body weight, which is poor nutritional status. In the article titled “Protein–Energy Malnutrition Is Associated with Worse Outcomes in Patients with Atrial Fibrillation: A Nationwide Analysis,” Markson et al.^1^ used a national database of 821,630 patients with AF hospitalizations between the years 2016–2017, including 21,385 (3%) with protein–energy malnutrition, which is defined by the presence of cachexia, kwashiorkor, marasmus, and other types of protein–calorie malnutrition (severe, unspecified). They found that protein–energy malnutrition is associated with worse in-hospital outcomes in patients with AF, including increases in mortality and the odds of cardiogenic shock and reduced odds of undergoing successful ablation and achieving the restoration of cardiac rhythm.

There is no obvious explanation for the lower success rate of ablation in patients with low body mass index (BMI) values, and more studies with adjusted analyses need to be conducted to better understand this phenomenon. What is more important, however, is the finding of a higher rate of complications of AF ablation in patients with poor nutritional status. This was demonstrated by a recent study by Tonegawa-Kujiout et al.^2^ that showed that having a low BMI of <18.5 kg/m^2^ was associated with an increased risk of cardiac tamponade when compared to having a normal BMI (18.5–25 kg/m^2^). Possible mechanisms for the increased risk of perforation in patients with low BMI values include greater relative levels of anticoagulation, smaller atria, and thinner atrial walls.

Poor nutrition and low BMI are not common among patients in developed countries, where most AF ablations are performed. However, patients with low BMI values are occasionally encountered, and it is important to realize that they are at a higher risk of complications than normal-weight patients during AF ablation, and, subsequently, a discussion with the patient and the care team is necessary prior to the procedure.

I hope that you enjoy reading the rest of this issue of *The Journal of Innovations in Cardiac Rhythm Management*.


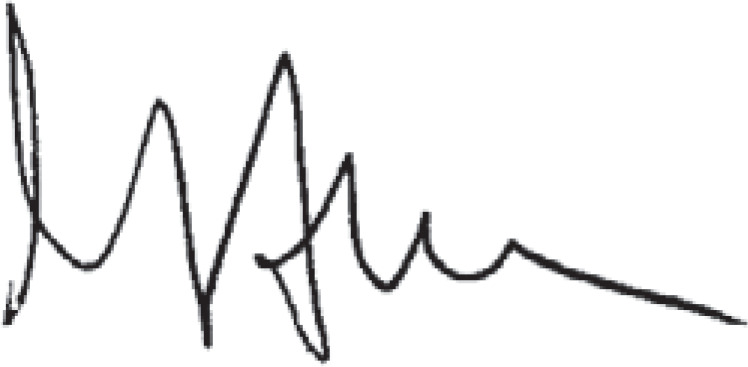


Sincerely,

Moussa Mansour, md, fhrs, facc

Editor in Chief

*The Journal of Innovations in Cardiac Rhythm Management*

MMansour@InnovationsInCRM.com

Director, Atrial Fibrillation Program

Jeremy Ruskin and Dan Starks Endowed Chair in Cardiology

Massachusetts General Hospital

Boston, MA 02114
